# Sharing leaky-integrate-and-fire neurons for memory-efficient spiking neural networks

**DOI:** 10.3389/fnins.2023.1230002

**Published:** 2023-07-31

**Authors:** Youngeun Kim, Yuhang Li, Abhishek Moitra, Ruokai Yin, Priyadarshini Panda

**Affiliations:** Department of Electrical Engineering, Yale University, New Haven, CT, United States

**Keywords:** spiking neural network, image recognition, event-based processing, energy-efficient deep learning, neuromorphic computing

## Abstract

Spiking Neural Networks (SNNs) have gained increasing attention as energy-efficient neural networks owing to their binary and asynchronous computation. However, their non-linear activation, that is Leaky-Integrate-and-Fire (LIF) neuron, requires additional memory to store a membrane voltage to capture the temporal dynamics of spikes. Although the required memory cost for LIF neurons significantly increases as the input dimension goes larger, a technique to reduce memory for LIF neurons has not been explored so far. To address this, we propose a simple and effective solution, EfficientLIF-Net, which shares the LIF neurons across different layers and channels. Our EfficientLIF-Net achieves comparable accuracy with the standard SNNs while bringing up to ~4.3× forward memory efficiency and ~21.9× backward memory efficiency for LIF neurons. We conduct experiments on various datasets including CIFAR10, CIFAR100, TinyImageNet, ImageNet-100, and N-Caltech101. Furthermore, we show that our approach also offers advantages on Human Activity Recognition (HAR) datasets, which heavily rely on temporal information. The code has been released at https://github.com/Intelligent-Computing-Lab-Yale/EfficientLIF-Net.

## 1. Introduction

Spiking Neural Networks (SNNs) have gained significant attention as a promising candidate for low-power machine intelligence (Wu et al., 2018, 2019; Roy et al., 2019; Fang et al., 2021a; Kundu et al., 2021; Christensen et al., 2022). By mimicking biological neuronal mechanisms, Leaky-Integrate-and-Fire (LIF) neurons in SNNs convey visual information with temporal binary spikes over time. The LIF neuron (Liu and Wang, 2001) considers temporal dynamics by accumulating incoming spikes inside a membrane potential, and generates output spikes when the membrane potential voltage exceeds a firing threshold. Such binary and asynchronous operation of SNNs incurs energy-efficiency benefits on low-power neuromorphic hardware (Furber et al., 2014; Akopyan et al., 2015; Davies et al., 2018; Orchard et al., 2021).

**Figure 1:**
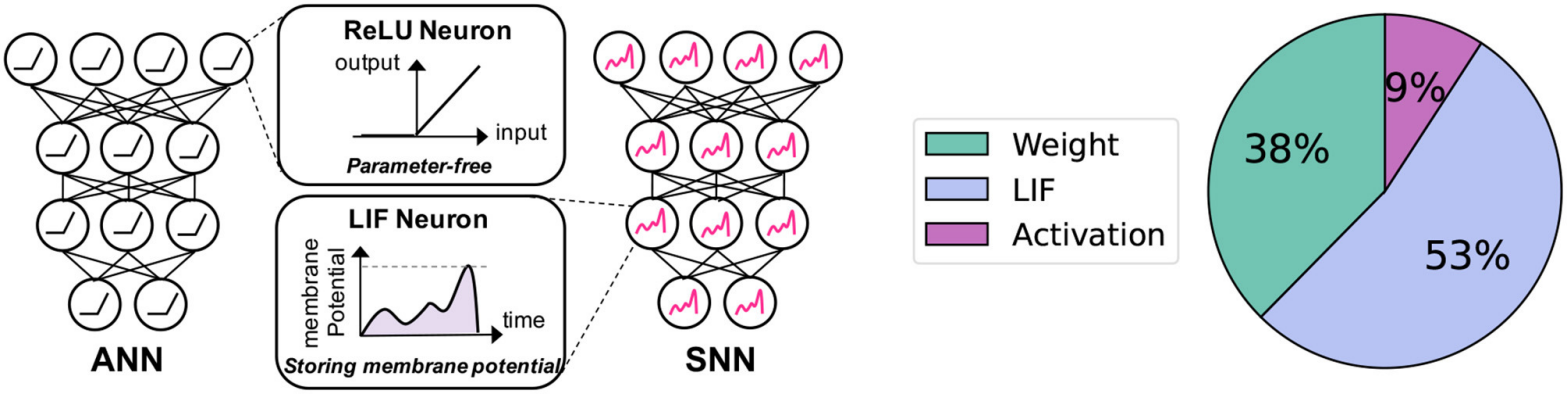
Motivation of our work. **Left:** Comparison between neurons in ANNs and SNNs: Unlike ReLU neurons, which do not require any parameters, LIF neurons maintain a membrane potential with voltage values that change across timesteps. **Right:** Memory cost breakdown for the Spiking-ResNet19 architecture during inference on an image with a resolution of 224 × 224.

Although SNN brings computational efficiency benefits, memory overhead caused by LIF neurons can be problematic. As shown in Figure 1, LIF neurons require additional memory for storing the membrane potential value which changes over time. This is not the case for the traditional Artificial Neural Networks (ANNs) where most non-linear activation functions are parameter-free (*e*.*g*.ReLU, Sigmoid). At the same time, LIF neurons occupy a large portion of memory with the high-resolution input image (Figure 1). For instance, the LIF memory takes 53% of memory overhead in the case of ResNet19 (He et al., 2016) with a 224 × 224 image. This analysis assumes 32-bit weight parameters, 1-bit spike activation, and 32-bit allocation for membrane potential. A more comprehensive analysis is provided in the “Memory Cost Break” subsection of Section 5.3.

Unfortunately, the LIF memory overhead has been overlooked so far in SNN studies.

To address this, we propose EfficientLIF-Net where we share the LIF neurons across different layers and channels. By sharing the memory, we do not need to assign separate memory for each layer and channel. For layer-wise sharing, we use common LIF neurons across layers having the same activation size, such as layers in one ResNet block (He et al., 2016). For channel-wise sharing, we equally divide the LIF neurons into multiple groups through the channel dimension and share common LIF neurons across different groups. Surprisingly, our EfficientLIF-Net provides similar performance as the standard SNN models where each layer and channel has independent LIF neurons. We show the gradient can successfully flow back through all layers, thus the weight can be trained to consider the temporal dynamics of spike information.

Furthermore, the proposed EfficientLIF-Net brings huge benefits to saving memory costs during training. Spatio-temporal operation inside SNNs incurs a huge computational graph for computing backward gradients. Each LIF neuron needs to store membrane potential to make gradients flow back, where the training memory increases as the SNN goes deeper and uses larger timesteps. This huge computational graph often is difficult to be trained on the limited GPU memory (Liang et al., 2021; Singh et al., 2022; Yin et al., 2022). In this context, since our architecture shares the membrane potential across all layers, we can compute each layer's membrane potential from the next layer's membrane potential real-time during backward step. This enables us to perform backpropagation without the need for storing/caching the membrane potentials of all layers in memory (from the forward step).

Our contributions can be summarized as follows:

- We pose the memory overhead problem of LIF neurons in SNNs, where the memory cost significantly increases as the image size goes larger.
- To address this, we propose a simple and effective architecture, EfficientLIF-Net where we share the LIF neurons across different layers and channels.
- EfficientLIF-Net also reduces memory cost during training by computing each layer's (channel's) membrane potential from the next layer's (channel's) membrane potential real-time during backward step, drastically reducing the caching of membrane potentials.
- We conduct experiments on five public datasets, validating EfficientLIF-Net can achieve comparable performance as the standard SNNs while bringing up to ~4.3× forward memory efficiency and up to ~21.9× backward memory efficiency for LIF neurons.
- We also observe that the LIF memory cost problem exists in pruned SNNs and in fact the LIF memory overhead percentage goes higher when the weight sparsity goes higher. Our EfficientLIF-Net successfully reduces the LIF memory cost to ~23% in pruned SNNs while achieving iso-accuracy compared to the pruned baseline.

## 2. Related work

### 2.1. Spiking neural networks

Different from the standard Artificial Neural Networks (ANNs), Spiking Neural Networks (SNNs) convey temporal spikes (Roy et al., 2019; Christensen et al., 2022). Here, Leaky-Integrate-and-Fire (LIF) neuron plays an important role as the non-linear activation. The LIF neurons have a “memory” called membrane potential, where the incoming spikes are accumulated. Output spikes are generated if the membrane potential exceeds a firing threshold, then the membrane potential resets to zero. This firing operation of LIF neurons is non-differentiable, so the previous SNN literature has focused on resolving the gradient problem. A widely-used training technique is converting pre-trained ANNs to SNNs using weight or threshold balancing (Diehl et al., 2015; Rueckauer et al., 2017; Sengupta et al., 2019; Han et al., 2020; Li et al., 2021a). However, such methods require large number of timesteps to emulate float activation using binary spikes. Recently, a line of works propose to circumvent the non-differentiable backpropagation problem by defining a surrogate function (Lee et al., 2016, 2020; Shrestha and Orchard, 2018; Wu et al., 2018, 2020, 2021; Neftci et al., 2019; Li et al., 2021b; Kim et al., 2022a). As the weight is trained to consider temporal dynamics, they show both high performance and short latency. Recent studies have expanded our understanding of Spiking Neural Networks (SNNs) and proposed novel approaches to overcome some of their inherent challenges. Hao et al. (2023) explores the issue of unevenness error in the conversion of ANNs to SNNs and introduces an optimization strategy based on residual membrane potential to mitigate this error, achieving state-of-the-art performance on several datasets. Li and Zeng (2022) examine the conversion of ANNs to SNNs from a different angle, addressing performance degradation and time delays. The authors propose a neuron model for releasing burst spikes and a novel method, Lateral Inhibition Pooling, to resolve inaccuracies caused by the MaxPooling operation during the conversion process. Che et al. (2022) present a spike-based differentiable hierarchical search (SpikeDHS) framework, providing efficient architecture search and training for SNNs. The authors also propose an innovative approach to optimize the surrogate gradient function, enhancing the performance of SNNs in various classification tasks. Lastly, paper (Guo et al., 2022a) introduces Information Maximization Loss (IM-Loss) to maximize the information flow in SNNs and proposes a novel differentiable spike activity estimation, Evolutionary Surrogate Gradients (ESG), which enhances both model convergence and task performance. Although the previous methods have made huge advances in terms of improving the performance, they assume that SNNs have different LIF neurons for different layers and channels, which imposes a huge memory overhead in both forward and backward.

### 2.2. Compression methods for efficient SNNs

Due to the energy-efficiency benefit of SNNs, they can be suitably implemented on edge devices with limited memory storage (Skatchkovsky et al., 2020; Venkatesha et al., 2021; Yang et al., 2022). Therefore, a line of work has proposed various methods to reduce the memory cost for SNNs using compression techniques. Neural pruning is one of the effective methods for SNN compression. Several works (Neftci et al., 2016; Rathi et al., 2018) have proposed a post-training pruning technique using a threshold value. Unsupervised online adaptive weight pruning (Guo et al., 2020) dynamically prunes trivial weights over time. Shi et al. (2019) prune weight connections during training with a soft mask. Recently, deeper SNNs are pruned with ADMM optimization tool (Deng et al., 2021), gradient-based rewiring (Chen et al., 2021), and lottery ticket hypothesis (Kim et al., 2022b). Meanwhile, various quantization techniques also have been proposed to compress SNNs (Datta et al., 2022; Guo et al., 2022b; Li et al., 2022a; Meng et al., 2022). Schaefer and Joshi (2020) propose integer fixed-point representations for neural dynamics, weights, loss, and gradients. The recent work (Chowdhury et al., 2021a) performs quantization through temporal dimension for low-latency SNNs. Lui and Neftci propose a quantization technique based on the Hessian of weights (Lui and Neftci, 2021). Nonetheless, no prior work has explicitly addressed the memory overhead caused by LIF neurons. Our method effectively reduces memory overhead by modifying the architecture, and is orthogonal to previous methods. Thus, combining EfficientLIF-Net with compression techniques will further compound the benefits.

## 3. Preliminaries

### 3.1. Leaky integrate-and-fire neuron

In our paper, we mainly address the memory cost from a Leaky-Integrate-and-Fire (LIF) neuron, which is widely adopted in SNN works (Wu et al., 2018, 2020, 2021; Lee et al., 2020; Fang et al., 2021a,b; Li et al., 2021a,b; Kim et al., 2022a). Suppose LIF neurons in *l*-th layer have membrane potential $U_{l}^{t}$ at timestep *t*, we can formulate LIF neuron dynamics as:


(1) $$\begin{array}{l} U_{l}^{t}=\lambda U_{l}^{t-1}+W_{l}O_{l-1}^{t}, \end{array}$$

where *W*_*l*_ is weight parameters in layer *l*, $O_{l-1}^{t}$ represents the spikes from the previous layer, λ is a decaying factor in the membrane potential. Note, we use uppercase letters for matrix notation. The LIF neuron generates an output spike $O_{l}^{t}$ when the membrane potential exceeds the firing threshold θ. Here, we define the spike firing function as:


(2) $$f(U_{l}^{t})=O_{l}^{t}=\{{}_{0\text{ otherwise}}^{1\text{ if }U_{l}^{t}>\theta }.$$

After firing, the membrane potential can be reset to zero (*i*.*e*.hard reset), or reduced by the threshold value (*i*.*e*.soft reset). Thus, a LIF neuron always stores the membrane potential to capture the temporal information of spikes. The memory cost for LIF neurons is proportional to the input image dimension, which poses a huge memory overhead for high-resolution data such as ImageNet (Deng et al., 2009).

### 3.2. Gradient backpropagation in SNNs

For the class probability prediction, we accumulate the final-layer activation across all timesteps, followed by the Softmax function. We apply cross-entropy loss *L* for training the weights parameters. The backward gradients are calculated in both spatial and time axis (Wu et al., 2018; Neftci et al., 2019) according to the chain rule:


(3) $$\begin{array}{l} \frac{\partial L}{\partial W_{l}}=\underset{t}{\sum }\left(\frac{\partial L}{\partial O_{l}^{t}}\frac{\partial O_{l}^{t}}{\partial U_{l}^{t}}+\frac{\partial L}{\partial U_{l}^{t+1}}\frac{\partial U_{l}^{t+1}}{\partial U_{l}^{t}}\right)\frac{\partial U_{l}^{t}}{\partial W_{l}}. \end{array}$$

Here, the gradient of output spikes with respect to the membrane potential $\frac{\partial O_{l}^{t}}{\partial U_{l}^{t}}$ is non-differentiable. Following previous work (Fang et al., 2021a), we use *arctan*() to approximate gradients, *i*.*e*.we use an approximate function $f\left(x\right)=\frac{1}{\pi }arctan\left(\pi x\right)+\frac{1}{2}$ for computing gradients of $\frac{\partial O_{l}^{t}}{\partial U_{l}^{t}}$. The overall computational graph is illustrated in **Figure 3A**.

## 4. Methodology: EfficientLIF-Net

**Figure 2:**
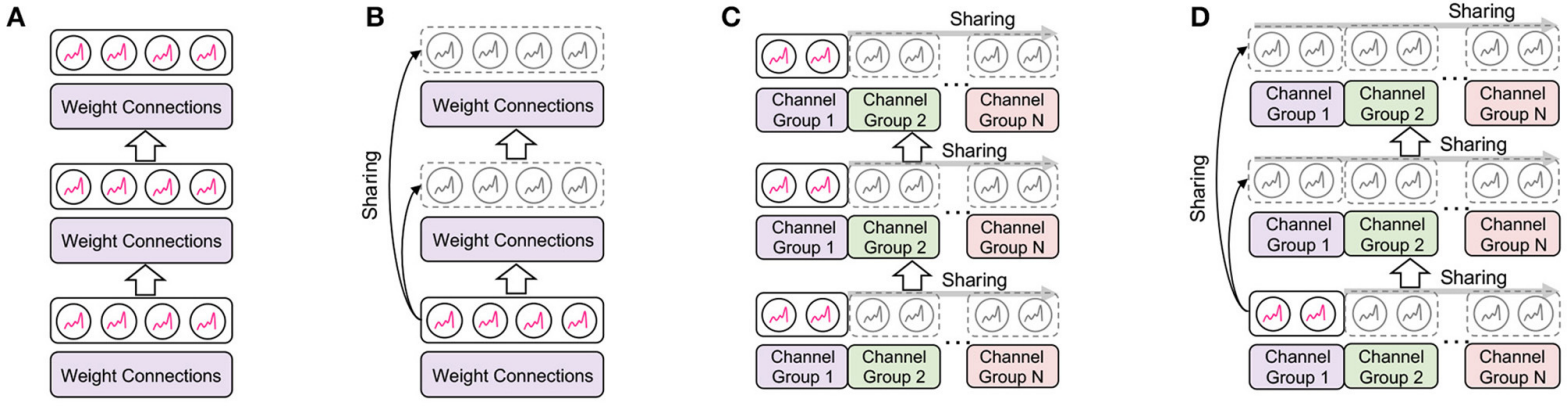
Illustration of the proposed EfficientLIF-Net. **(A)** Conventional SNNs where each layer and channel has separate LIF neurons. **(B–D)** is our proposed EfficientLIF-Net which shares LIF neurons across layer, channel, and layer & channel. **(A)** Baseline SNN. **(B)** Cross-layer sharing. **(C)** Cross-channel sharing. **(D)** Cross-layer & channel sharing.

In this section, we first describe the details of how we reduce the memory cost of LIF neurons across layers and channels. The overall concept of EfficientLIF-Net is illustrated in Figure 2. After that, we provide the analysis of the backward gradient in EfficientLIF-Net for training, which shows our EfficientLIF-Net successfully considers the entire time horizon. Finally, we show the memory advantage of our EfficientLIF-Net during backpropagation.

### 4.1. Sharing memory of LIF neurons

#### 4.1.1. Cross-layer sharing

The key idea here is sharing the LIF neurons across different layers where they have the same output activation size. Thus, LIF neurons are shared across multiple subsequent layers before the layer increases channel size or reduces spatial resolution. Such architecture design can be easily observed in CNN architectures such as ResNet (He et al., 2016).

Let's assume the networks have the same activation size from the *l*-th layer to the (*l*+*m*)-th layer. The membrane potential of the (*l*+1)-th layer is calculated by adding the previous layer's membrane potential and weighted spike output from the previous layer:


(4) $$\begin{array}{l} U_{l+1}^{t}=\lambda \left(U_{l}^{t}-O_{l}^{t}\right)+W_{l+1}O_{l}^{t}. \end{array}$$

Here the previous layer's membrane potential $U_{l}^{t}$ decreases its value by the threshold for soft reset (firing threshold is set to 1) after it generates spikes $O_{l}^{t}$. After that, decay factor λ is applied to the previous layer's membrane potential, since we aim to dilute the previous layers' information as networks go deeper. The layer (*l*+1) generates output spike following Eq. 2:


(5) $$\begin{array}{l} O_{l+1}^{t}=f\left(U_{l+1}^{t}\right). \end{array}$$

**Figure 3:**
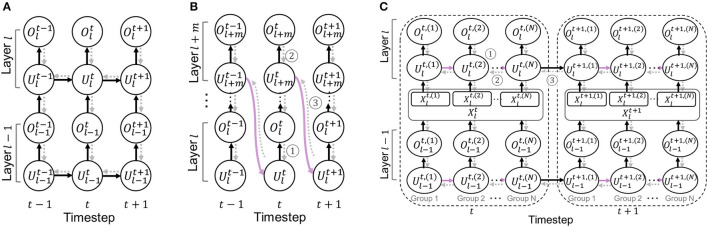
Illustration of an unrolled computational graph for the backpropagation. Black solid arrows and gray dotted arrows represent forward and backward paths, respectively. For simplicity, we omit the reset path from the spike output. **(A)** Baseline SNN. **(B)** Cross-layer sharing. **(C)** Cross-channel sharing.

In the same timestep, the spike information goes through all layers (from *l*-th layer to *l*+*m*-th layer) with Eqs. 4 and 5 dynamics. Then, the membrane potential of layer *l*+*m* is shared with layer *l* at the next timestep (purple arrow in Figure 3B).


(6) $$\begin{array}{l} U_{l}^{t+1}=\lambda \left(U_{l+m}^{t}-O_{l+m}^{t}\right)+W_{l}O_{l-1}^{t+1}, \end{array}$$

where the soft reset and decaying is applied to $U_{l+m}^{t}$, and the weighted input comes from layer *l*−1.

Overall, we require only one-layer LIF memory for layer *l*~ layer (*l*+*m*) computation, which is shared across all layers and timesteps. Thus, LIF memory of layers *l*~(*l*+*m*) can be reduced by $\frac{1}{m}$. The overall computational graph is illustrated in Figure 3B.

#### 4.1.2. Cross-channel sharing

We also explore the neuron sharing scheme in the channel dimension. Let *X*_*l*_ be the weighted input spike, *i*.*e*. $X_{l}=W_{l}O_{l-1}^{t}$, then we first divide the weighted input spike tensor into *N* groups in channel axis.


(7) $$\begin{array}{l} X_{l}^{t}\to \left[X_{l}^{t,\left(1\right)},X_{l}^{t,\left(2\right)},...,X_{l}^{t,\left(N\right)}\right]. \end{array}$$

Suppose $X_{l}^{t}\in {\mathbb{R}}^{C\times H\times W}$, then the spike of each group can be represented as $X_{l}^{t,\left(i\right)}\in {\mathbb{R}}^{\frac{C}{N}\times H\times W}$, *i*∈{1, 2, …, *N*}. Here, *C, H, W* represent the size of channel, height, and width, respectively. Then, the LIF neurons can be sequentially shared across different groups (*i*.*e*.different channels) of weighted input spike. The membrane potential of (*i*+1)-th group at layer *l* can be formulated as:


(8) $$\begin{array}{l} U_{l}^{t,\left(i+1\right)}=\lambda \left(U_{l}^{t,\left(i\right)}-O_{l}^{t,\left(i\right)}\right)+X_{l}^{t,\left(i+1\right)}, \end{array}$$

where $U_{l}^{t,\left(i\right)}$ is the membrane potential of the previous group, and $X_{l}^{t,\left(i+1\right)}$ is the incoming weighted spike input of the (*i*+1)-th group from the previous layer. Here, soft reset and decaying also applied. The output spikes of each group are generated by standard firing dynamics (Eq. 2):


(9) $$\begin{array}{l} O_{l}^{t,\left(i\right)}=f\left(U_{l}^{t,\left(i\right)}\right). \end{array}$$

We concatenate the output spikes of each groups through channels in order to compute the output at timestep *t*:


(10) $$\begin{array}{l} O_{l}^{t}=\left[O_{l}^{t,\left(1\right)},O_{l}^{t,\left(2\right)},...,O_{l}^{t,\left(N\right)}\right]. \end{array}$$

After completing the LIF sharing in timestep *t*, we share the last group's (*i*.*e*.group *N*) membrane potential to the first group in the next timestep *t*+1.


(11) $$\begin{array}{l} U_{l}^{t+1,\left(1\right)}=\lambda \left(U_{l}^{t,\left(N\right)}-O_{l}^{t,\left(N\right)}\right)+X_{l}^{t+1,\left(1\right)}. \end{array}$$

By using cross-channel sharing, the memory cost for LIF neuron of one layer can be reduced by $\frac{1}{N}$, where *N* is the number of groups. Thus, memory-efficiency will increase as we use larger group number.

#### 4.1.3. Cross-layer & channel sharing

The cross-layer and cross-channel sharing methods are complementary to each other, therefore they can be used together to bring further memory efficiency. The LIF neurons are shared across channels and layers as shown in Figure 2D. The neuron-sharing mechanism can be obtained by combining cross-layer and cross-channel sharing methods.

Let's assume the networks have the same activation size from the *l*-th layer to the (*l*+*m*)-th layer. The sharing mechanism in one layer is the same as channel sharing (Eq. 7 ~ 9).

Thus, the output spikes of each group through channels in order to compute the output at timestep *t*:


(12) $$\begin{array}{l} O_{l}^{t}=\left[O_{l}^{t,\left(1\right)},O_{l}^{t,\left(2\right)},...,O_{l}^{t,\left(N\right)}\right]. \end{array}$$

After completing the LIF sharing at layer *l*, we share the last group's (*i*.*e*.group *N*) membrane potential of *l*-th layer to the first group of *l*+1-th layer.


(13) $$\begin{array}{l} U_{l+1}^{t,\left(1\right)}=\lambda \left(U_{l}^{t,\left(N\right)}-O_{l}^{t,\left(N\right)}\right)+X_{l+1}^{t,\left(1\right)}. \end{array}$$

Here, $X_{l}^{t}$ stands for the weighted input spike, *i*.*e*. $X_{l}^{t}=W_{l}O_{l-1}^{t}$. In the same timestep, the spike information goes through all layers (from *l*-th layer to *l*+*m*-th layer) dynamics. Then, the last group's (*i*.*e*.group *N*) membrane potential of layer *l*+*m* is shared with the first group of layer *l* at the next timestep.


(14) $$\begin{array}{l} U_{l}^{t+1,\left(1\right)}=\lambda \left(U_{l+m}^{t,\left(N\right)}-O_{l+m}^{t,\left(N\right)}\right)+X_{l}^{t+1,\left(1\right)}. \end{array}$$

By using cross-channel sharing, the memory cost of LIF neuron for layer *l*~ layer (*l*+*m*) computation can be reduced by $\frac{1}{mN}$, where *N* is the number of groups. Our experimental results show that although we combine two sharing methods, we still get iso-accuracy as the standard SNNs.

### 4.2. Gradient analysis

Sharing LIF neurons leads to different gradient paths compared to standard SNNs. Therefore, we provide the gradient analysis for EfficientLIF-Net.

#### 4.2.1. Gradient of cross-layer sharing

Suppose that we compute the gradients for *m* subsequent layers where they have the same activation size. For simplicity, we call these *m* subsequent layers as a “sharing block”. The unrolled computational graph is illustrated in Figure 3B.

For the intermediate layers of the sharing block, the gradients flow back from the next layer (marked as ➀ in Figure 3B), which can be formulated as:


(15) $$\begin{array}{l} \frac{\partial L}{\partial W_{l}}=\underset{t}{\sum }\left(\frac{\partial L}{\partial O_{l}^{t}}\frac{\partial O_{l}^{t}}{\partial U_{l}^{t}}+\frac{\partial L}{\partial U_{l+1}^{t}}\frac{\partial U_{l+1}^{t}}{\partial U_{l}^{t}}\right)\frac{\partial U_{l}^{t}}{\partial W_{l}}, \end{array}$$

where both terms are derived by the forward dynamics in Eq. 4. For the final layer of the sharing block, the gradients flow back through both layer and temporal axis:


(16) $$\begin{array}{l} \frac{\partial L}{\partial W_{l+m}}=\underset{t}{\sum }\left(\frac{\partial L}{\partial O_{l+m}^{t}}\frac{\partial O_{l+m}^{t}}{\partial U_{l+m}^{t}}+\frac{\partial L}{\partial U_{l}^{t+1}}\frac{\partial U_{l}^{t+1}}{\partial U_{l+m}^{t}}\right)\frac{\partial U_{l+m}^{t}}{\partial W_{l+m}}. \end{array}$$

The first term shows the gradient from the next layer (marked as ➁ in Figure 3B), and the second term is from the first layer of the sharing block at the next timestep (marked as ➂ in Figure 3B). The last layer of the sharing block obtains the gradients from the next timestep (marked as ➂) which is then, propagated through the intermediate layers. This allows the weight parameters to be trained with temporal information, achieving similar performance as the standard SNN architecture.

#### 4.2.2. Gradient of cross-channel sharing

Assume that we divide the channel into *N* groups. We define an index set *G* = {1, 2, …, *N*}. Then, the gradients of weight parameters in layer *l* can be computed as:


(17) $$\begin{array}{l} \frac{\partial L}{\partial W_{l}}=\sum_{t}\sum_{i\in G}\frac{\partial L}{\partial O_{l}^{t,(i)}}\frac{\partial O_{l}^{t,(i)}}{\partial U_{l}^{t,(i)}}\frac{\partial U_{l}^{t,(i)}}{\partial X_{l}^{t}}\frac{\partial X_{l}^{t}}{\partial W_{l}}\\ \;+\sum_{t}\sum_{i\in G\backslash \{N\}}\frac{\partial L}{\partial U_{l}^{t,(i+1)}}\frac{\partial U_{l}^{t,(i+1)}}{\partial U_{l}^{t,(i)}}\frac{\partial U_{l}^{t,(i)}}{\partial X_{l}^{t}}\frac{\partial X_{l}^{t}}{\partial W_{l}}\\ \;+\sum_{t}\frac{\partial L}{\partial U_{l}^{t+1,(1)}}\frac{\partial U_{l}^{t+1,(1)}}{\partial U_{l}^{t,(N)}}\frac{\partial U_{l}^{t,(N)}}{\partial X_{l}^{t}}\frac{\partial X_{l}^{t}}{\partial W_{l}}. \end{array}$$

The first term represents the gradient from the next layer (marked as ➀ in Figure 3C). The second term is the gradients from the next group's membrane potential except for the last group (marked as ➁ in Figure 3C). The last term represents the gradients from the first group of the next timestep (marked as ➂ in Figure 3C). Thus, the gradients propagate through both temporal and spatial dimension, training weight parameters to consider the temporal information.

### 4.3. Memory-efficient backpropagation

**Figure 4:**
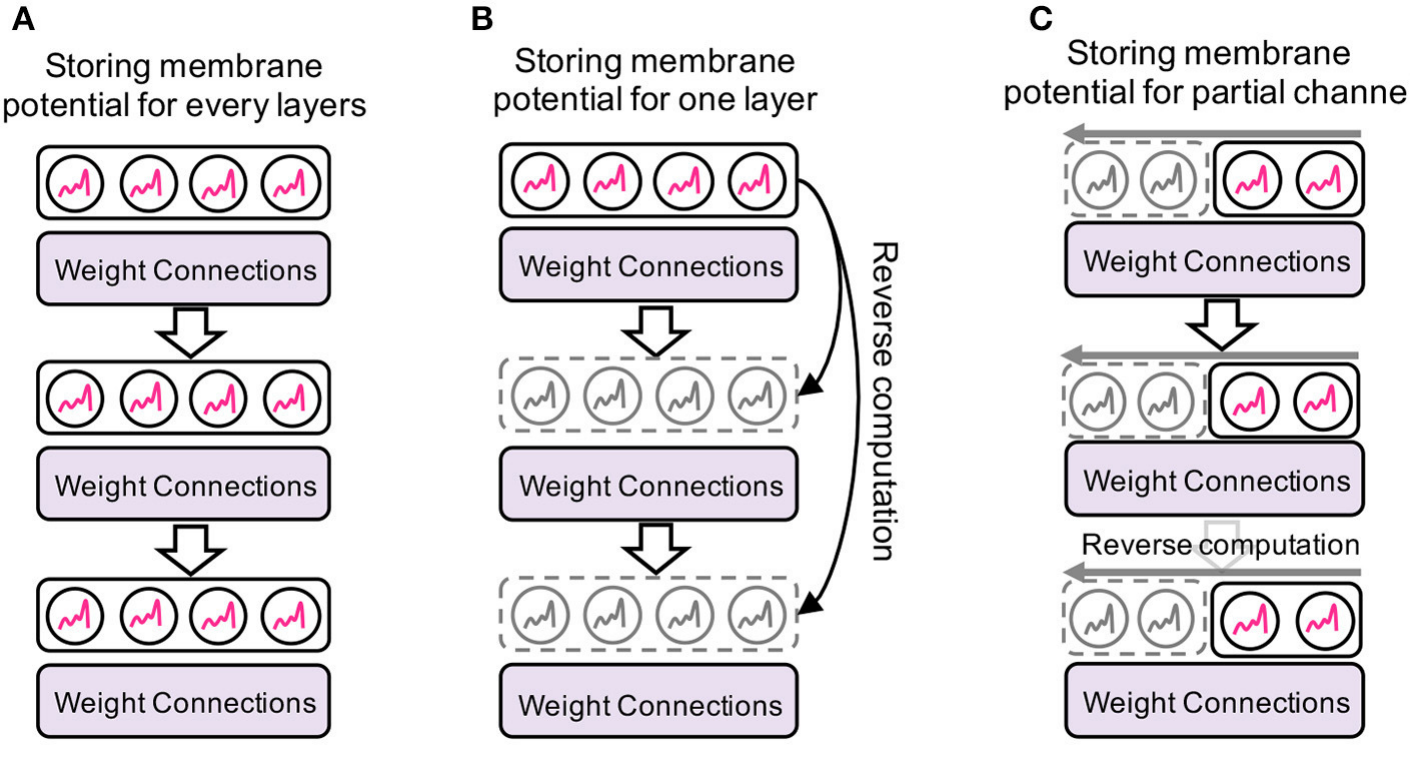
Memory-efficient backpropagation. Compared to baseline, we do not need to store an intermediate membrane potential for backpropagation. Instead, we perform a reverse computation on the membrane potential from the next layers/channels. **(A)** Baseline. **(B)** Cross-layer. **(C)** Cross-channel.

In addition to the memory efficiency in forward propagation, our EfficientLIF-Net saves memory costs during backward gradient computation. As shown in Figure 4A, the standard SNNs need to store all membrane potential to compute the gradient such as $\frac{\partial U_{l}^{t+1}}{\partial U_{l}^{t}}$ in Eq. 3. However, saving the full-precision membrane potential of LIF neurons is costly.

#### 4.3.1. Backpropagation in cross-layer sharing

The key idea here is that the membrane potential of the previous layer can be computed from the next layer's membrane potential in a reverse way (Figure 4B). Thus, without storing the membrane potential of the intermediate layers during forward, we can compute the backward gradient. By reorganizing Eqs. 4 and 6, we obtain the membrane potential of the previous layer or the previous timestep.


(18) $$\{{}_{U_{l+m}^{t}=\frac{1}{\lambda }(U_{l}^{t+1}-W_{l}O_{l-1}^{t+1})+O_{l+m}^{t}.\text{ from Eq. 6}}^{U_{l}^{t}=\frac{1}{\lambda }(U_{l+1}^{t}-W_{l+1}O_{l}^{t})+O_{l}^{t}.\text{ from Eq. 4}}$$

Based on this, we can compute $\frac{\partial U_{l+1}^{t}}{\partial U_{l}^{t}}$ in Eq. 15, and $\frac{\partial U_{l}^{t+1}}{\partial U_{l+m}^{t}}$ in Eq. 16, without storing the intermediate membrane potential.

#### 4.3.2. Backpropagation in cross-channel sharing

In a similar way, we can also reduce memory cost through channel dimension by performing a reverse computation on the membrane potential of channel groups (Figure 4C). Instead of storing a memory for all channels, we use a partial memory for storing the membrane potential of the last group channel of each layer. From Eq. 8 and 11, we calculate the membrane potential of the previous channel group or the previous timestep.


(19) $$\{{}_{U_{l}^{t,(N)}=\frac{1}{\lambda }(U_{l}^{t+1,(1)}-X_{l}^{t+1,(1)})+O_{l}^{t,(N)}.\text{ from Eq}.\;11}^{U_{l}^{t,(i)}=\frac{1}{\lambda }(U_{l}^{t,(i+1)}-X_{l}^{t,(i+1)})+O_{l}^{t,(i)}.\text{ from Eq. 8}}$$

This reverse computation allows us to compute $\frac{\partial U_{l}^{t,\left(i+1\right)}}{\partial U_{l}^{t,\left(i\right)}}$ and $\frac{\partial U_{l}^{t+1,\left(1\right)}}{\partial U_{l}^{t,\left(N\right)}}$ in Eq. 17, without storing the intermediate membrane potential.

Note that our approach does not modify the structure of the backpropagation graph. Instead, it optimizes memory usage by replacing previous memory values with those required for gradient computation. After calculating the gradient for a specific timestep or layer, it becomes unnecessary to maintain the intermediate variables. This strategy enables us to repurpose that memory for future gradient computations, creating a more hardware-efficient solution.

### 4.4. Hardware discussion

In this section, we aim to provide insights into the role that EfficientLIF-Net will play during hardware deployment.

#### 4.4.1. Cross-layer Sharing

Cross-layer sharing EfficientLIF-Net can largely benefit hardware implementation with reduction of memory communication. When deploying an SNN on hardware, one can either choose to process through all the layers and then repeat for all timesteps (standard) or first process through all timesteps and then proceed to the next layer [tick-batch (Narayanan et al., 2020)]. While the tick-batch can help to reduce the number of memory communication across timesteps, it requires more hardware resources. On the other hand, with a proper processing pipeline across layers, the standard way of processing SNNs will have smaller hardware resource requirement and larger throughput. And cross-layer sharing can further reduce the memory communication overhead of the standard SNN processing.

**Figure 5:**
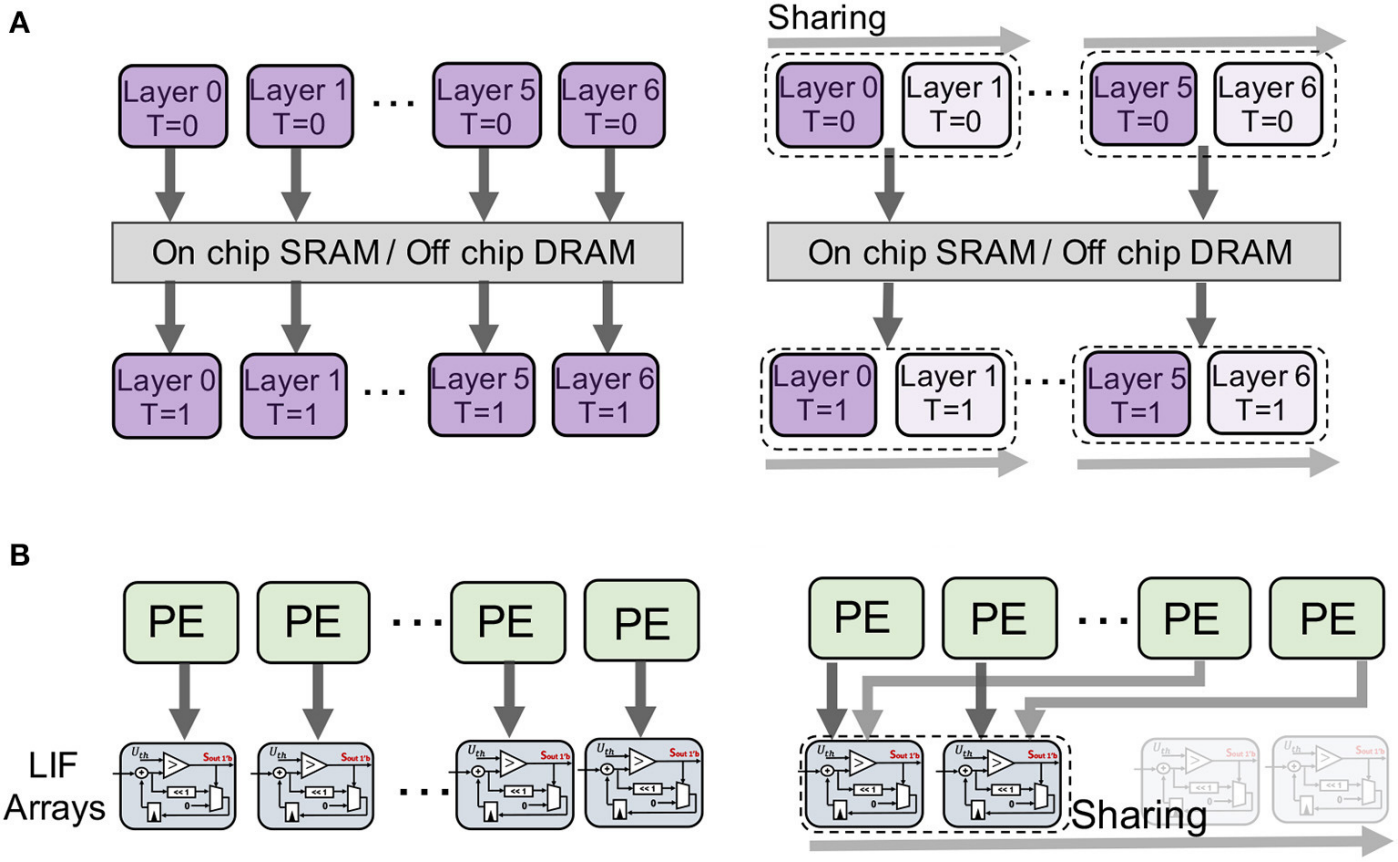
Visualization of the potential hardware mapping of the two sharing methods. We provide some hardware insights on the potential hardware benefits we can get from the EfficientLIF-Net. **(A)** Cross-layer sharing. **(B)** Cross-channel sharing.

As we show in Figure 5A, instead of writing the membrane potential to the memory for every layer and every timestep, layer-sharing EfficientLIF-Net requires only one time of writing to memory for each shared layer for each timestep.

#### 4.4.2. Cross-channel Sharing

Due to the high level of parallelism and data reuse in these designs, we are focusing on examining the effects of cross-channel sharing on EfficientLIF-Net for ASIC systolic array-based inference accelerators for SNNs (Narayanan et al., 2020; Lee et al., 2022; Yin et al., 2022). The key idea behind this group of designs is to broadcast input spikes and weights to an array of processing elements (PEs), where accumulators perform convolution operations. Each post-synaptic neuron's entire convolution operation is mapped to one dedicated PE. Once the convolution results are ready, they are sent to the LIF units inside the PE to generate the output spikes. LIF units are notorious for their high hardware overheads. This is because we need at least one buffer to hold the full precision membrane potential for each neuron. These buffers heavily contribute to the hardware cost of LIF units. Originally, all the prior designs (Narayanan et al., 2020; Lee et al., 2022; Yin et al., 2022) equipped each of the PEs with an LIF unit inside to match the design's throughput requirements. That means, for 128 PE array, we will need 128 LIF units. Even if the number of LIF units is reduced, there is no way to reduce the number of buffers required to hold the unique membrane potentials for each LIF neuron.

Based on this design problem, we can instantly realize one advantage that cross-channel sharing EfficientLIF can bring in these hardware platforms. Depending on the number of cross-channel shared LIF neurons, we can have the same ratio of LIF units and buffer reduction at the hardware level, as we show in Figure 5B. For example, in the case of C#4 shared networks, we can manage to reduce the 128 LIF units in a 128 PE array (Narayanan et al., 2020; Lee et al., 2022; Yin et al., 2022) to 32. However, the shared LIF units will bring longer latency as a trade-off. In the case of C#4, originally, one cycle was needed to generate spikes from 128 post-synaptic neurons for one timestep. Now, we will need 4 cycles instead. However, the major portion of latency still lies in the convolution and memory operations, which is typically hundreds of times larger than the cycles needed for generating spikes through LIF units. We provide experimental results in Section V.C to further illustrate the effects of EfficientLIF-Net on hardware.

## 5. Experiments

### 5.1. Implementation details

We evaluate our method on four static image datasets [*i*.*e*.CIFAR10 (Krizhevsky et al., 2009), CIFAR100 (Krizhevsky et al., 2009), TinyImageNet (Deng et al., 2009), ImageNet-100 (Deng et al., 2009)], and one spiking dataset [*i*.*e*.N-Caltech101(Orchard et al., 2015)]. Here, ImageNet-100 is the subset of ImageNet-1000 dataset (Deng et al., 2009). We use VGG16 (Simonyan and Zisserman, 2015) and ResNet19 (He et al., 2016). For both architectures, we use the scaled-up channel size following previous SNN works (Zheng et al., 2020; Li et al., 2022b). We train the SNNs with 128 batch samples using SGD optimizer with momentum 0.9 and weight decay 5e-4. The initial learning rate is set to 0.1 and decayed with cosine learning rate scheduling (Loshchilov and Hutter, 2016). We set the total number of epochs to 300 for CIFAR10, CIFAR100, and N-Caltech101, and 140 for TinyImageNet and ImageNet-100, respectively. We use timesteps *T* = 5 across all experiments.

### 5.2. Performance comparison

Across all experiment sections, *EfficientLIF-Net[L]* denotes the cross-layer sharing scheme, *EfficientLIF-Net[C#N]* stands for the cross-channel sharing scheme with *N* channel groups. *EfficientLIF-Net[L+C#N]* means the cross-layer & channel sharing method.

In Table 1, we show the memory benefit from EfficientLIF-Net. We assume a 32-bit representation for membrane potential in LIF neurons. Regarding the backward LIF memory of baseline, we consider the standard backpropagation method which stores membrane potential across entire timesteps (Liang et al., 2021; Singh et al., 2022; Yin et al., 2022).

**Table 1:** Accuracy and LIF memory cost (Forward & Backward) comparison between baseline (*i*.*e*.standard SNN) and our EfficientLIF-Net.

	**VGG16**			
**Dataset**	**Methods**	**Acc (*%*)**	**LIF forward memory (MB)**	**LIF backward memory (MB)**
CIFAR10	Baseline	91.31	1.80	9.0
	EfficientLIF-Net [L]	90.23	1.23	1.23
	EfficientLIF-Net [C#2]	90.30	0.90	0.90
	EfficientLIF-Net [L+C#2]	90.09	0.62	0.62
CIFAR100	Baseline	66.83	1.80	9.0
	EfficientLIF-Net [L]	65.01	1.23	1.23
	EfficientLIF-Net [C#2]	64.92	0.90	0.90
	EfficientLIF-Net [L+C#2]	64.85	0.62	0.62
TinyImageNet	Baseline	56.11	7.22	36.1
	EfficientLIF-Net [L]	55.14	4.91	4.91
	EfficientLIF-Net [C#2]	55.43	3.61	3.61
	EfficientLIF-Net [L+C#2]	55.36	2.46	2.46
ImageNet-100	Baseline	73.81	88.43	442.15
	EfficientLIF-Net [L]	73.22	60.10	60.10
	EfficientLIF-Net [C#2]	72.65	44.21	44.21
	EfficientLIF-Net [L+C#2]	72.14	30.05	30.05
N-Caltech101	Baseline	64.40	4.06	40.6
	EfficientLIF-Net [L]	63.50	2.76	2.76
	EfficientLIF-Net [C#2]	64.02	2.03	2.03
	EfficientLIF-Net [L+C#2]	63.10	1.38	1.38
	**ResNet19**			
**Dataset**	**Methods**	**Acc (**%**)**	**LIF forward memory (MB)**	**LIF backward memory (MB)**
CIFAR10	Baseline	92.26	2.88	14.40
	EfficientLIF-Net [L]	91.99	1.31	1.31
	EfficientLIF-Net [C#2]	91.92	1.44	1.44
	EfficientLIF-Net [L+C#2]	91.73	0.66	0.66
CIFAR100	Baseline	70.89	2.88	14.40
	EfficientLIF-Net [L]	70.14	1.31	1.31
	EfficientLIF-Net [C#2]	70.01	1.44	1.44
	EfficientLIF-Net [L+C#2]	69.99	0.66	0.66
TinyImageNet	Baseline	56.74	11.5	57.5
	EfficientLIF-Net [L]	55.20	5.25	5.25
	EfficientLIF-Net [C#2]	55.44	5.75	5.75
	EfficientLIF-Net [L+C#2]	55.10	2.63	2.63
ImageNet-100	Baseline	79.38	140.88	704.4
	EfficientLIF-Net [L]	79.44	64.31	64.31
	EfficientLIF-Net [C#2]	78.92	70.44	70.44
	EfficientLIF-Net [L+C#2]	78.88	32.16	32.16
N-Caltech101	Baseline	66.27	6.47	64.7
	EfficientLIF-Net [L]	65.82	2.95	2.95
	EfficientLIF-Net [C#2]	66.01	3.24	3.24
	EfficientLIF-Net [L+C#2]	65.45	1.48	1.48

Here, EfficientLIF-Net[L], EfficientLIF-Net[C#2], EfficientLIF-Net[L+C#2] denote EfficientLIF with cross-layer, cross-channel (#group=2), and cross-layer & channel sharing, respectively.

The experimental results show the following observations: (1) The EfficientLIF-Net based on ResNet19 achieves a similar performance compared to the baseline, which implies that the proposed membrane sharing strategy still can learn temporal information in spikes. (2) The EfficientLIF-Net also can be applied to the DVS dataset. (3) The ResNet19 EfficientLIF-Net achieves less performance degradation compared to VGG16, which implies that skip connection improves training capability in EfficientLIF-Net. Furthermore, ResNet19 brings higher memory efficiency since it has more layers with similar sized activation. (4) As expected, a large-resolution image dataset has more benefits compared to a small-resolution image dataset. For instance, *EfficientLIF-Net [L+C#2]* saves 108.72 MB and 672.24 MB for forward and backward path, respectively, on ImageNet-100 which consists of 224 × 224 resolution images, on the other hand, the same architecture saves 2.22 MB (forward) and 13.74 MB (backward) on CIFAR10. Here, we report the theoretical calculations. Our results imply the membrane potential is not as important for SNNs. This observation has been presented in the previous work Chowdhury et al. (2021b); Li et al. (2023b,a) where they show SNN can work with very low timestep 1~2.

Note that the approaches to memory reduction proposed by other works, such as those reducing simulation time step Chowdhury et al. (2021b) and reducing SNN time dependence Meng et al. (2023), can be combined with our layer/channel-wise sharing technique. This would lead to an even more significant decrease in memory usage, demonstrating the compatibility and potential of our method when integrated with other optimization strategies.

### 5.3. Experimental analysis

#### 5.3.1. Analysis on training dynamics

**Figure 6:**
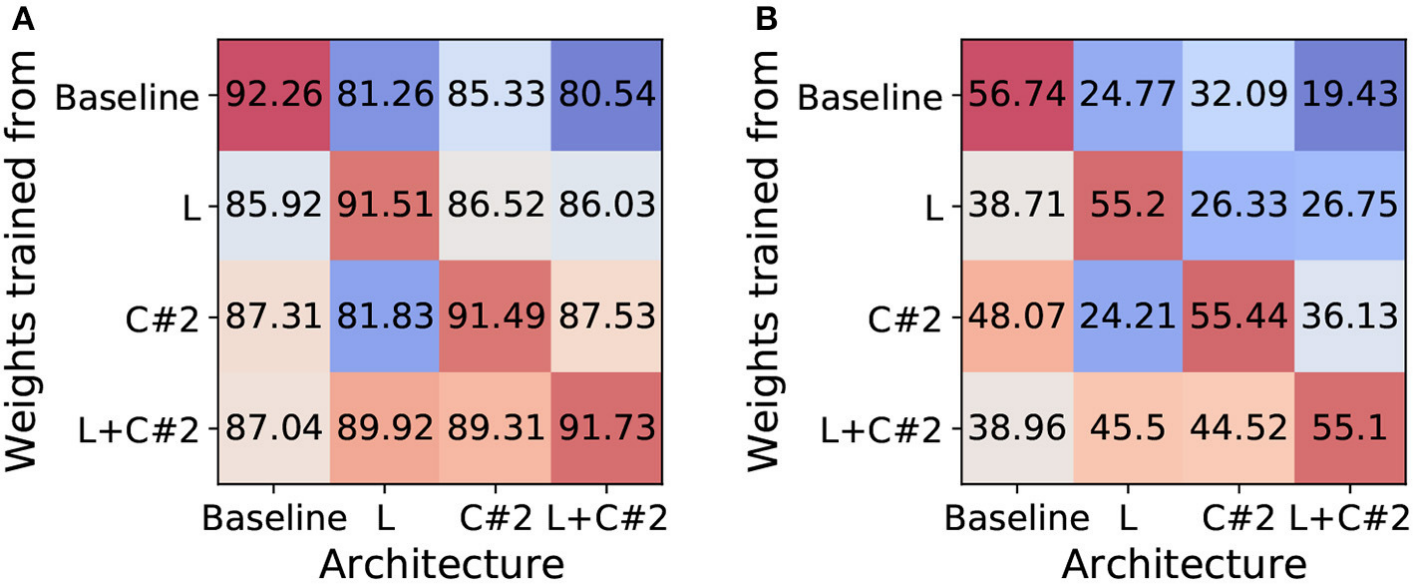
Analysis on Training Dynamics. Unit: accuracy (%). We investigate whether the trained weight parameters can be compatible with other architectures. **(A)** CIFAR10. **(B)** TinyImageNet.

In our method section, we showed that the backward gradients of each method are different. To further analyze this, we investigate whether the trained weight parameters can be compatible with other architectures. We expect that the transferred weights to different architectures may show performance degradation since each architecture has different training dynamics (*e*.*g*.gradient path). To this end, we train standard ResNet19-SNN (*i*.*e*.baseline), EfficientLIF-Net [L], EfficientLIF-Net [C#2], and EfficientLIF-Net [L+C#2], In Figure 6, we report the accuracy of various weights-architecture configurations on CIFAR10 and TinyImageNet. We observe the following points: (1) As we expected, transferring weights to a different architecture brings performance degradation. This supports our statement that each architecture has different training dynamics. (2) Especially, baseline shows a huge performance drop as compared to other architectures. Thus, EfficientLIF-Net needs to be trained from scratch with gradient-based training. (3) The trained weights from EfficientLIF-Net [L+C#2] show reasonable performance on EfficientLIF-Net [L] and EfficientLIF-Net [C] as it contains the feature from both cross-layer and cross-channel sharing.

#### 5.3.2. Ablation studies on #group

In the cross-layer sharing scheme, we can further reduce LIF memory cost by increasing *#group*. Table 2 shows the accuracy and LIF memory cost with respect to *#group*. Interestingly, EfficientLIF-Net with high *#group* almost maintains the performance while minimizing the LIF memory cost significantly. For example, on the ImageNet-100 dataset, EfficientLIF-Net [C#8] incurs only 0.8% accuracy drop with 75% higher memory saving. Thus, one can further reduce LIF memory cost by increasing *#group* based on the hardware requirements. We hypothesize that the observed decrease in performance could be attributed to the mixing of information across channels during sharing. It is a widely recognized phenomenon in the field of neural networks that the preservation of discriminative representation across channels is crucial for optimal performance. However, when we share membrane potential across channels, subsequent groups may be influenced by information from prior groups due to the sequential nature of this sharing process. While we have suggested a potential cause, we aim to delve deeper into this issue in our future research.

**Table 2:** Ablation on the number of groups in cross-channel EfficientLIF-Net with ResNet19 architecture.

**Dataset**	**Methods**	**Acc (*%*)**	**LIF memory for Fw & Bw (MB)**
CIFAR10	EfficientLIF-Net [C#2]	91.92	1.44
	EfficientLIF-Net [C#4]	91.73	0.72
	EfficientLIF-Net [C#8]	91.21	0.36
TinyImageNet	EfficientLIF-Net [C#2]	55.44	5.75
	EfficientLIF-Net [C#4]	55.06	2.88
	EfficientLIF-Net [C#8]	54.84	1.44
ImageNet-100	EfficientLIF-Net [C#2]	78.92	70.44
	EfficientLIF-Net [C#4]	78.24	35.22
	EfficientLIF-Net [C#8]	78.12	17.61

#### 5.3.3. Combining with group convolution

To further enhance the efficiency in cross-channel sharing, we explore the feasibility of combining a group convolution layer with cross-layer sharing. Since group convolution splits input channels and output channels into multiple groups, they can be applied to each channel spike ($O_{l}^{t,\left(i\right)}$ in Eq. 10). In Table 3, we observe the accuracy does not show a huge drop with two convolution groups. However, as the number of groups increases, the performance goes down drastically due to lesser number of parameters available for training convergence.

**Table 3:** Performance of combing cross-layer sharing and group convolution on ResNet 19 architecture.

**Dataset**	**Methods**	**#Conv. group**	**Acc (*%*)**
CIFAR10	EfficientLIF-Net [C#2]	2	91.42
	EfficientLIF-Net [C#4]	4	90.45
	EfficientLIF-Net [C#8]	8	87.38
CIFAR100	EfficientLIF-Net [C#2]	2	69.26
	EfficientLIF-Net [C#4]	4	66.42
	EfficientLIF-Net [C#8]	8	60.20
TinyImageNet	EfficientLIF-Net [C#2]	2	53.65
	EfficientLIF-Net [C#4]	4	51.39
	EfficientLIF-Net [C#8]	8	42.86
N-Caltech	EfficientLIF-Net [C#2]	2	64.98
	EfficientLIF-Net [C#4]	4	60.94
	EfficientLIF-Net [C#8]	8	55.12

#### 5.3.4. Soft reset vs. hard reset

We also conduct experiments on the reset scheme in our EfficientLIF-Net. The membrane potential can be reset to zero (*i*.*e*.hard reset), or decreased by the threshold value (*i*.*e*.soft reset). In Table 4, we compare the accuracy of both reset schemes on ResNet19 architecture, where we observe the hard reset achieves similar accuracy as the soft reset. However, using the hard reset does not allow reverse computation of the previous layer's or timestep's membrane potential (Eq. 18 and 19) during backpropagation. This is because the hard reset removes the residual membrane potential which can be used in the reverse computation. Therefore, our EfficientLIF-Net is based on the soft reset such that we get memory savings both during forward and backward.

**Table 4:** Ablation on the reset methods.

**Dataset**	**Methods**	**Reset scheme**	**Acc (*%*)**
CIFAR10	EfficientLIF-Net [L]	Soft	91.99
	EfficientLIF-Net [L]	Hard	91.66
	EfficientLIF-Net [C#2]	Soft	91.92
	EfficientLIF-Net [C#2]	Hard	91.67
	EfficientLIF-Net [L+C#2]	Soft	91.73
	EfficientLIF-Net [L+C#2]	Hard	91.65
CIFAR100	EfficientLIF-Net [L]	Soft	70.14
	EfficientLIF-Net [L]	Hard	70.05
	EfficientLIF-Net [C#2]	Soft	70.01
	EfficientLIF-Net [C#2]	Hard	68.93
	EfficientLIF-Net [L+C#2]	Soft	69.99
	EfficientLIF-Net [L+C#2]	Hard	69.74
N-Caltech	EfficientLIF-Net [L]	Soft	65.82
	EfficientLIF-Net [L]	Hard	64.44
	EfficientLIF-Net [C#2]	Soft	66.01
	EfficientLIF-Net [C#2]	Hard	64.98
	EfficientLIF-Net [L+C#2]	Soft	65.45
	EfficientLIF-Net [L+C#2]	Hard	64.12

#### 5.3.5. Analysis on spike rate

**Figure 7:**
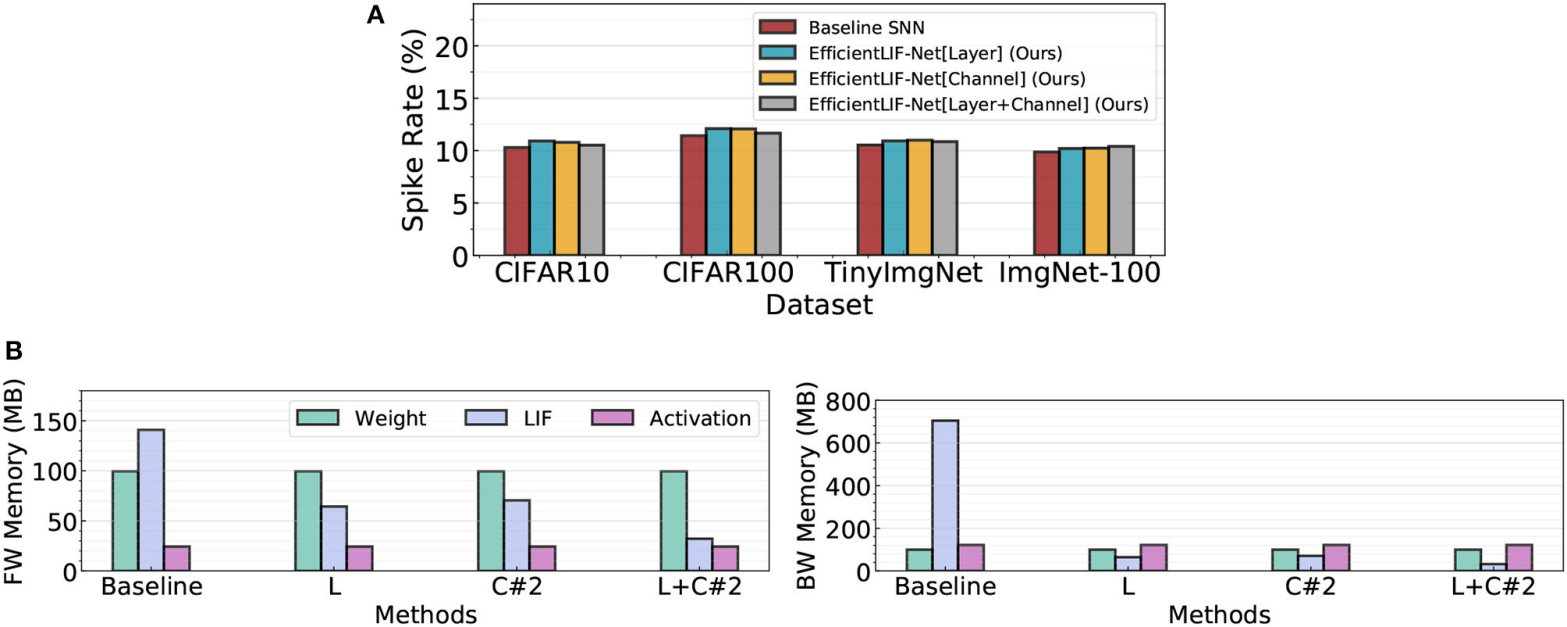
**(A)** Spike rate analysis on four public datasets. **(B)** Comparison of the memory breakdown between the baseline SNN and the EfficientLIF-Net in both forward and backward. We use ResNet19 architecture on ImageNet-100.

In Figure 7A, we compare the spike rate across all different LIF sharing schemes in ResNet19. We conduct experiments on four datasets. Note, a high spike rate implies the networks require larger computational cost. The experimental results show that all LIF sharing schemes have a similar spike rate as the baseline. This demonstrates that EfficientLIF-Net does not bring further computational overhead while saving memory cost by sharing the membrane potential.

#### 5.3.6. Time overhead analysis

We measured the time overhead on a V100 GPU with a batch size of 128. We used VGG16 with CIFAR10 and ImageNet-100 datasets with image sizes of 32 × 32 and 224 × 224, respectively. Table 5 shows the latency results for each method. Interestingly, we found that our method improves computation time, implying that our LIF layer-sharing method reduces the time required to access DRAM, which originally takes a significant percentage of computational time. As a result, our method can be implemented without a huge computational burden.

**Table 5:** Analysis on the computational time.

**Method (latency: ms)**	**32 × 32**	**224 × 224**
Baseline	105.12	148.21
EfficientLIF-Net [L]	79.21	131.25
EfficientLIF-Net [C#2]	80.62	142.75
EfficientLIF-Net [L+C#2]	81.26	143.05

#### 5.3.7. Memory cost breakdown

In Figure 7B, we compare the memory cost breakdown between the SNN baseline and EfficientLIF-Net in both forward and backward. In the memory cost comparison, we consider memory for weight parameters (32-bit), spike activation (1-bit), and LIF neurons (32-bit). In the baseline SNN, LIF neurons take a dominant portion for both forward and backward memory cost. Especially, for backward, LIF neurons occupy around 7× larger memory than weights or activation memory. Our EfficientLIF-Net significantly reduces the LIF memory cost, resulting in less memory overhead compared to weight parameters (in both forward and backward) and activation (in backward only).

#### 5.3.8. EfficientLIF-Net with weight pruning

As pruning for SNNs is popular due to its usage on edge devices (Neftci et al., 2016; Shi et al., 2019; Guo et al., 2020; Chen et al., 2021; Kim et al., 2022b), it is important to figure out whether the advantage from EfficientLIF-Net remains in sparse SNNs.

**Figure 8:**
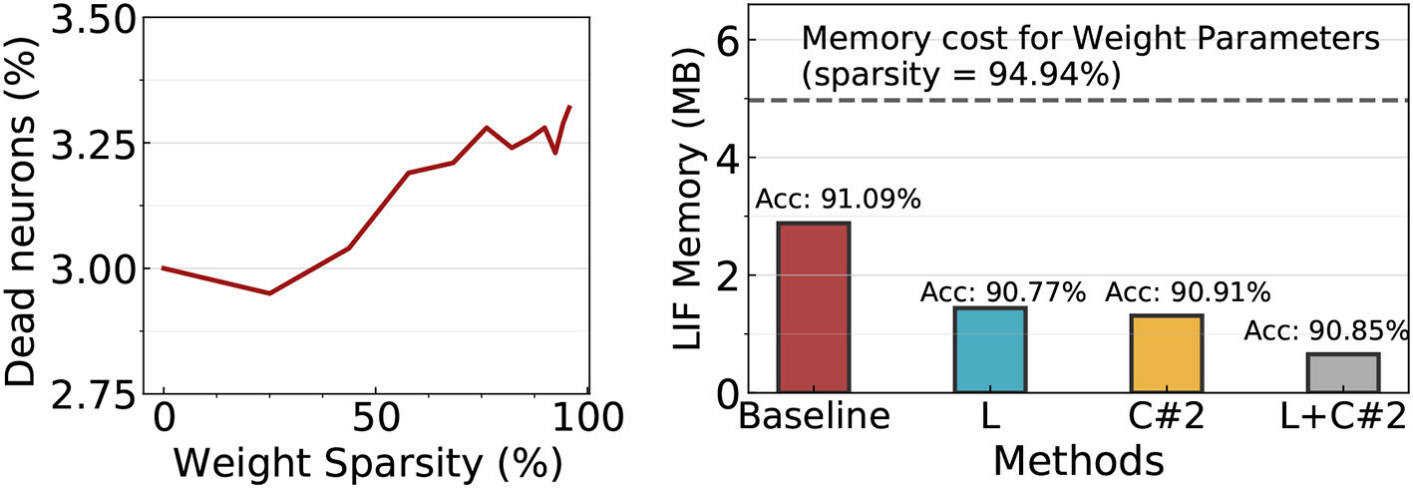
Experiments on ResNet19 EfficientLIF-Net with weight pruning methods on CIFAR10. **Left:** Most LIF neurons generate output spikes although the weight sparsity increases. Therefore, the LIF memory cost cannot be reduced by weight pruning. **Right:** Accuracy and LIF memory cost comparison across baseline and EfficientLIF-Net. The weight memory cost across all models is ~5*MB* indicated with a gray dotted line.

Before exploring the effectiveness of the LIF sharing method in sparse SNNs, we first investigate if LIF neurons still require a huge memory in sparse SNNs. This is because a number of LIF neurons might not generate output spikes in the high weight sparsity regime (≥90%), then, the memory cost for such dead neurons can be reduced. To this end, we prune the SNN model to varied sparsity using magnitude-based pruning (Han et al., 2015). Interestingly, as shown in Figure 8 Left, only ~3% neurons do not generate spikes (*i*.*e*.dead neuron) across all sparsity levels. This implies that the LIF memory cost is still problematic in sparse SNNs. Based on the observation, we prune EfficientLIF-Net and compare the memory cost and accuracy with the standard SNN baseline. Here, we prune all architectures to have 94.94% weight sparsity. In Figure 8 Right, the baseline architecture requires 2.9 MB for LIF neurons, which is equivalent to ~60% of the memory cost for weight parameters. With cross-layer (denoted as *L* in Figure 8) and cross-channel sharing (denoted as *C#2* in Figure 8), we can reduce the LIF memory cost by about half compared to the baseline. Cross-layer & channel sharing (denoted as *L+C#2* in Figure 8) further reduces the memory cost, which takes only ~23% memory compared to the baseline. Overall, the results demonstrate that LIF memory reduction is not only important for high-resolution images but also for relatively low-resolution images such as CIFAR10 especially when considering pruned SNNs.

#### 5.3.9. Hardware evaluation

**Figure 9:**
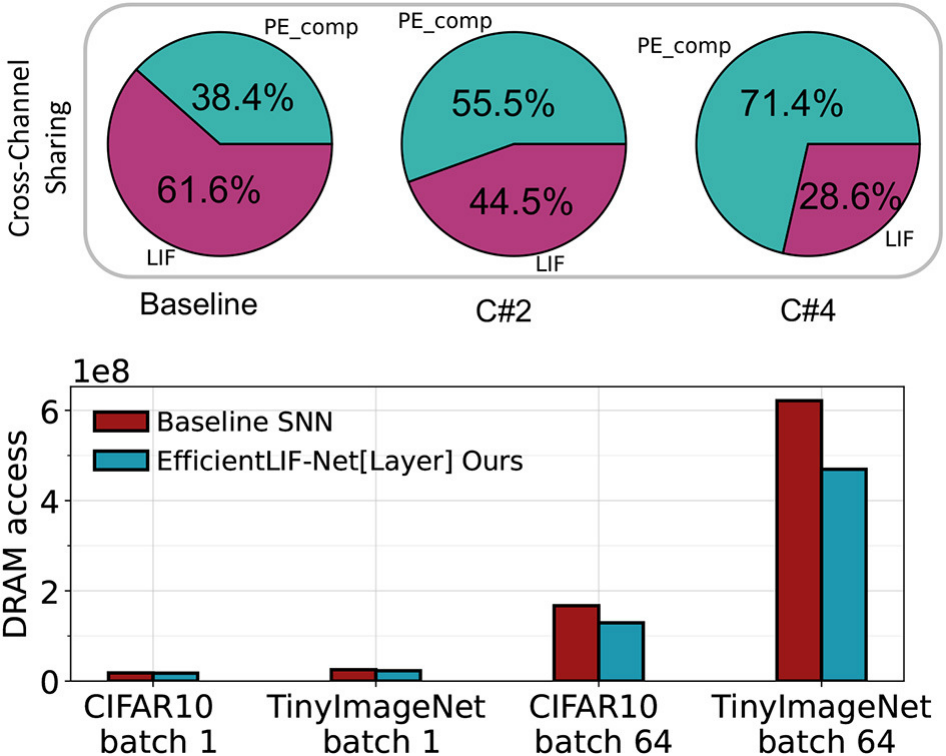
**Top**: The breakdown of computation for the Baseline SNN, EfficientLIF-Net[C#2], and EfficientLIF-Net[C#4] in a 128 PE array implemented on SATA . **Bottom**: Comparison of DRAM access reduction between the Baseline SNN and EfficientLIF-Net[Layer] on VGG-16 across various datasets. The reduction is contrasted for single batch processing and multiple mini-batch processing scenarios.

As discussed in Section 4.4, both cross-channel and cross-layer sharing can significantly enhance hardware efficiency during deployment. From the top portion of Figure 9, it is evident that cross-channel sharing in EfficientLIF-Net can considerably decrease the number of required LIF units. Specifically, our approach reduces the compute requirement of LIF units inside the PE from 61.6 to 28.6% of the total PE computation when employing C#4 cross-channel sharing.

The bottom part of Figure 9 indicates that cross-layer sharing can effectively minimize the number of DRAM accesses, which is the most energy-consuming operation during on-chip SNN inference. For single-batch scenarios, the reduction is not significant, since weight data movement dominates the DRAM accesses, as outlined in (Yin et al., 2022). However, when employing mini-batches, the reduction becomes more substantial. We note a 23 and 25% reduction in total DRAM accesses on CIFAR10 and TinyImageNet, respectively, for 64 mini-batches. This reduction trend continues to rise with larger mini-batch numbers.

### 5.4. Evaluation on human activity recognition datasets

To further validate our method on datasets that rely heavily on temporal information, we conduct experiments using Human Activity Recognition (HAR) datasets obtained from wearable devices. Descriptions of these datasets are provided below:

- *UCI-HAR* (Anguita et al., 2013) consists of 10.3 k instances collected from 30 subjects, involving six different activities: walking, walking upstairs, walking downstairs, sitting, standing, and lying. The dataset employs sensors such as a 3-axis accelerometer and a 3-axis gyroscope (both at 50Hz) from a Samsung Galaxy SII.
- *HHAR* (Stisen et al., 2015) is collected from nine subjects and encompasses six daily activities: biking, sitting, standing, walking, stair ascent, and stair descent. The dataset utilizes accelerometers from eight smartphones and four smartwatches (with sampling rates ranging from 50 to 200 Hz).

Following previous work, we split both datasets into 64% for the training set, 16% for the validation set, and 20% for the test set. We report test accuracy when the model achieves its best validation accuracy.

In Table 6, we compare our method with the baseline model, which consists of six 1D-convolutional layers, *i*.*e*., *Conv*1*D*(*InputChannel*, 128)−4 × *Conv*1*D*(128, 128)−*Conv*1*D*(128, *#Class*). In addition, we provide the performance of other methods (Avilés-Cruz et al., 2019; Mukherjee et al., 2020; Wang and Liu, 2020) on HHAR and UCI-HAR. Avilés-Cruz et al. (2019) uses a CNN, Mukherjee et al. (2020) uses a combination of CNN and LSTM, and Wang and Liu (2020) uses an LSTM. From the table, we can observe the following results: (1) The baseline Spiking MLP achieves an accuracy of 97.68% on the HHAR dataset and 96.06% on the UCI-HAR dataset, which is comparable accuracy with the previous methods. (2) Comparing the different configurations of EfficientLIF-Net to the baseline Spiking MLP, we can see that the EfficientLIF-Net maintains a similar level of accuracy as the baseline on both datasets. These results suggest that our LIF-sharing method also works well with tasks that heavily rely on temporal information. Overall, our empirical results support the observation that gradients propagate through both temporal and spatial dimensions, effectively training the weight parameters to account for temporal information, as demonstrated in Eq. 15, 16, and 17.

**Table 6:** Accuracy (%) comparison between baseline (*i*.*e*., 6 layer 1D-Convolutional SNN) and our EfficientLIF-Net.

**Method/dataset**	**HHAR (Stisen et al., 2015)**	**UCI-HAR (Anguita et al., 2013)**
Avilés-Cruz et al. (2019)	96.19	96.29
Mukherjee et al. (2020)	97.15	97.87
Wang and Liu (2020)	95.59	82.41
Spiking MLP (Baseline)	97.68	96.06
EfficientLIF-Net [L]	97.10	95.58
EfficientLIF-Net [C#2]	97.68	96.06
EfficientLIF-Net [L+C#2]	97.10	95.04

Here, EfficientLIF-Net[L], EfficientLIF-Net[C#2], EfficientLIF-Net[L+C#2] denote EfficientLIF with cross-layer, cross-channel (#group=2), and cross-layer & channel sharing, respectively.

## 6. Conclusion

In this paper, we highlight and tackle the problem of LIF memory cost in SNNs. This problem becomes severe as the image resolution increases. To address this, we propose EfficientLIF-Net where we share the membrane potential across layers and channels, which can effectively reduce memory usage. During backpropagation, our EfficientLIF-Net also enables reverse computation on the previous layer and channel. Therefore, we only need to store the membrane potential of the last layer/channel during forward. In our experiments, EfficientLIF-Net achieves similar performance and computational cost while significantly reducing memory cost compared to standard SNN baseline. We also found that the LIF memory problem exists in sparse-weight SNNs where even a small resolution dataset causes LIF memory overhead. The memory benefit of EfficientLIF-Net is shown in pruned SNNs, which implies our method is complementary to previous compression methods.

## Data availability statement

The original contributions presented in the study are included in the article/supplementary material, further inquiries can be directed to the corresponding author.

## Author contributions

YK and PP conceived the work. YK, YL, AM, and RY carried out experiments. YK, RY, and PP contributed to the writing of the paper. All authors contributed to the article and approved the submitted version.
